# Maternal oxygen exposure may not change umbilical cord venous partial pressure of oxygen: non-random, paired venous and arterial samples from a randomised controlled trial

**DOI:** 10.1186/s12884-020-03212-3

**Published:** 2020-09-04

**Authors:** Yunhai Chuai, Wen Jiang, Xiaobin Xu, Aiming Wang, Yuanqing Yao, Lei Chen

**Affiliations:** 1 Chinese PLA General Hospital, Medical School of Chinese PLA, Fuxing Road No. 28, Beijing, 100853 China; 2 Department of Obstetrics and Gynaecology, Sixth Medical Center, Chinese PLA General Hospital, Beijing, China; 3 Department of Anaesthesia, Chaoyang Chinese Traditional and Western Medicine Emergency Medical Center, Beijing, China; 4 Department of Obstetrics and Gynaecology, First Medical Center, Chinese PLA General Hospital, Beijing, China

**Keywords:** Low flow oxygen, The second stage of labor, Umbilical cord venous PO_2_

## Abstract

**Background:**

Despite the widespread use of oxygen (O_2_) in intrauterine resuscitation, the obstetric scientists’ understanding of O_2_ therapy is full of contradictions. We tested the hypothesis that higher maternal arterial partial pressure of oxygen (PO_2_) is associated with higher umbilical cord venous PO_2_ (UvPO_2_).

**Methods:**

This is a planned secondary analysis of a randomised controlled trial (RCT), 443 normal women were 1:1 randomly allocated to receive 2 L/min O_2_ or room air from the onset of second stage to delivery. We reported that maternal 2 L/min O_2_ exposure cannot affect the umbilical cord arterial pH or the fetal heart rate (FHR) pattern. In 217 non-random samples, we found 2 L/min O_2_ exposure increased the maternal arterial PO_2_ to the median 150 mmHg (hemoglobin would be saturated). The primary outcome for this analysis was UvPO_2_ in these non-random samples.

**Results:**

There were no significant differences between the O_2_ group (*N* = 107) and the control group (*N* = 110) in the UvPO_2_ (median 30.2, interquartile 25.4–35.2 versus median 28.3, interquartile 23.4–35.3, mmHg, *P* = 0.379). There were also no significant differences between room air and different percentiles of O_2_ exposure duration (< 25th, ≧ 25th < 50th, ≧ 50th < 75th, ≧ 75th percentile) in the UvPO_2_.

**Conclusions:**

Maternal O_2_ exposure at super-physiological levels (median arterial blood PO_2_ 150 mmHg) in normal labor may not change the UvPO_2_.

**Clinical trial registration:**

ClinicalTrials.govNCT02221440, first posted in 20 August 2014.

## Background

Oxygen (O_2_) therapy is widely used in pregnant women. It is estimated that more than 50% of births with normal oxygenation are given additional O_2_ [1]. Obstetricians and midwives use O_2_ to improve suspicious fetal heart monitoring patterns or fetal acid-base metabolism. However, the obstetric scientists’ understanding of O_2_ therapy is full of contradictions. The American College of Obstetricians and Gynecologists (ACOG) pointed out the lack of evidence for maternal O_2_ in Practice Bulletin 106 issued in 2009 [2], based on a Cochrane systematic review [3], the Cochran systematic review stated that there was not enough evidence that maternal prophylactic or therapeutic O_2_ inhalation could improve fetal acid-base metabolism; however, ACOG supported maternal O_2_ in Practice Bulletin 116 issued in 2010 [4]. Based on a cohort study of 56 people without a control group [5], the conclusion was reached that 10 L/min high-flow mask O_2_ inhalation could effectively improve the oxygenation status of the fetus. The Society for Maternal-Fetal Medicine has also published a continuous discussion on maternal O_2_ administration with no consensus [1, 6–9]. Maternal O_2_ therapy has been written into the latest textbook for obstetrics and gynecology in China [10]. However, this textbook did not provide specific references, nor did it specify the medical indications, concentration or flow range, oxygenation duration, or efficacy of O_2_ therapy.

In 2017, our trial randomized 443 normal women at the beginning of second stage to get nasal cannula O_2_ at 2 L/min or room air. There were no differences between O_2_ group and control group in the umbilical cord arterial pH (7.261 versus 7.266), and the study found partial pressure of oxygen (PO_2_) (mmHg) of woman was statistical higher in the O_2_ group than that of control group (150.0 versus 112.0 mmHg) [11]. The present study was a planned secondary analysis of our randomised controlled trial (RCT). Maternal arterial blood PO_2_ is about 100–110 mmHg in room air; when PO_2_ reaches about 150 mmHg, maternal hemoglobin will be saturated; and when PO_2_ exceeds 150 mmHg, O_2_ that continues to increase can only be transported by means of physical dissolution [12]. The purpose of the paper was to evaluate the effect of maternal O_2_ administration (radial arterial blood PO_2_ median 150 and interquartile 142.6–156.7 mmHg) in the second stage on the umbilical venous PO_2_ (UvPO_2_), the primary outcome was the effect of O_2_ exposure on UvPO_2_ and the second outcome was the effect of duration of O_2_ exposure on UvPO_2_.

## Methods and participants

This study was carried out at the Sixth Medical Center, Chinese PLA General Hospital, from September 2014 to May 2015 in Beijing, Chin, institutional Review Board Number was 08/29/2014. The trial was first posted in clinicaltrials.gov (NCT02221440) 20 August 2014. This manuscript reported adherence to CONSORT guidelines.

The trial included 443 women who had no obstetric complications with category I fetal heart rate (FHR) drawings at the first stage and normal labor at the beginning of second stage. FHR was reviewed as recommended by the ACOG Practice Bulletin category I (normal), category II (indeterminate), category III (abnormal) (Table S1) [2, 4]. Labor was managed according to a consensus from Chinese Medical Association [13]. Participants were 1:1 randomly assigned to get either 2 L/min O_2_ or sham at 0 L/min O_2_ by the nasal cannula from the onset of second stage to delivery. Participants, investigators, and outcomes assessors were blinded to the contents of the nasal cannulas until the conclusion of the study. We assessed the umbilical cord arterial pH and the FHR pattern from all 443 participants, and reported maternal 2 L/min O_2_ exposure cannot affect the umbilical cord arterial pH or the FHR pattern [11].

All women were invited to participate in radial arterial blood gas analysis, and radial arterial blood samples were obtained in 217 women who voluntarily agreed (these radial samples were non-randomly obtained), paired umbilical cord venous and arterial samples that were also obtained in these participants by one trained research nurse. We selected results from paired arterial and venous samples if venous-arterial pH difference > 0.02. The primary outcome for this analysis was UvPO_2_ (mmHg). These were compared between women with different O_2_ exposure durations, defined as <25th, ≧ 25th < 50th, ≧ 50th < 75th, ≧ 75th percentile of the duration of exposure in O_2_ group, respectively. Outcomes were also compared between room air and different percentiles of O_2_ exposure duration.

Statistical analysis was performed by the SPSS (IBM SPSS Statistics 22.0). To analyze normal distribution of continuous variables, we used the Kolmogorov-Smirnov test. To analyze normal distributed variables, we used the Student’s *t* test. To analyze abnormal distributed variables, we used the Mann-Whitney *U* test. Kruskal-Wallis *H* test was used to determine differences between two or more groups. Data were expressed as median (interquartile range) or mean ± standard deviation. *P* < 0.05 was statistical significance.

## Results

The Fig. S1 showed the recruitment and follow-up of the study.

The Table 1 showed the baseline characteristics of these non-random samples. Of the 443 patients were randomly assigned, 217 participants provided radial arterial blood samples and paired umbilical cord venous and arterial samples, there were 107 patients who were assigned to receive nasal cannula O_2_ at 2 L/min and 110 who were assigned to sham administered. The baseline characteristics were same between O_2_ group and control group.

**Table 1:** Baseline characteristics

Outcome	Oxygen Group (***n*** = 107)	Placebo Group (***n*** = 110)	***P***
Age (y)	30 (27–32)	29 (28–31)	0.62
Gestational age (wk)	40.0 (39.3–40.9)	40.0 (39.3–40.4)	0.22
Admission BMI (kg/m^2^)	25.8 (24.5–27.6)	26.6 (24.5–28.6)	0.16
Antepartum hemoglobin (g/L)	127.1 [9.1]	127.2 [9.8]	0.93
Duration of first stage (min)	450 (310–590)	450 (300–660)	0.76
Duration of second stage (min)	47 (31–67)	42 (28–58)	0.29
Maternal pH	7.367 (7.337–7.391)	7.359 (7.332–7.381)	0.16
Maternal PO_2_ (mmHg)	150.0 (142.6–156.7)	112.0 (104.8–118.3)	< 0.001
Maternal PCO_2_ (mmHg)	26.1 [4.1]	27.2 [3.4]	0.03

Data are expressed as median (25th–75th percentile) or mean [standard deviation]

*BMI* body mass index

Data from reference 11

Venous-arterial pH differences were > 0.02 in all paired arterial and venous samples. There were no differences between the two groups in the umbilical venous pH (median 7.349, interquartile 7.297–7.397 versus median 7.353, interquartile 7.301–7.381, *P* = 0.361), the PO_2_ (median 30.2, interquartile 25.4–35.2 versus median 28.3, interquartile 23.4–35.3, mmHg, *P* = 0.379), or the PCO_2_ (median 41.8, interquartile 36.1–47.5 versus median 42.6, interquartile 35.3–50.7, mmHg, *P* = 0.39). There were also no differences between the two groups in the umbilical arterial PO_2_ (median 18.6, interquartile 15.7–23.2 versus median 19.3, interquartile 14.6–24.7, mmHg, *P* = 0.299), or the PCO_2_ (median 54.5, interquartile 44.8–66.7 versus median 58, interquartile 48.3–65.6, mmHg, *P* = 0.19). (Table 2).

**Table 2:** Paired venous and arterial umbilical cord blood gases

Outcome	Oxygen Group (***n*** = 107)	Placebo Group (***n*** = 110)	***P***
Umbilical venous gas			
pH	7.349 (7.297–7.397)	7.353 (7.301–7.381)	0.361
PO_2_ (mmHg)	30.2 (25.4–35.2)	28.3 (23.4–35.3)	0.379
PCO_2_ (mmHg)	41.8 (36.1–47.5)	42.6 (35.3–50.7)	0.39
Umbilical arterial gas			
p H[11]	7.264 (7.232–7.299)	7.266 (7.217–7.298)	0.7
PO_2_ (mmHg)	18.6 (15.7–23.2)	19.3 (14.6–24.7)	0.299
PCO_2_ (mmHg)	54.5 (44.8–66.7)	58 (48.3–65.6)	0.19

Data are expressed as median (25th–75th percentile)

There were also no differences between different percentiles of O_2_ exposure duration (*N* = 26, < 25th; *N* = 27, ≧ 25th < 50th; N = 27, ≧ 50th < 75th; N = 27, ≧ 75th percentile) and room air in the UvPO_2_ (*P* = 0.681). (Table 3).

**Table 3:** The effect of duration of oxygen exposure on umbilical cord venous PO_2_

Outcomes	Air (***N*** = 110)	Percentiles of oxygen exposure duration				***P***
		<25th (***N*** = 26)	≧25th < 50th (***N*** = 27)	≧50th < 75th (***N*** = 27)	≧75th (***N*** = 27)	
**Umbilical cord venous PO**_**2**_ **(mmHg)**	28.3 (23.4–35.3)	30.3 (25.7–35.1)	27.4 (25.3–34.2)	31.6 (27.7–35.5)	29.4 (23.2–34.8)	0.681
**Maternal radial arterial PO**_**2**_ **(mmHg)**	112.0 (104.8–118.3)	150.4 (142.7–156.3)	151.8 (142.8–158.8)	150 (146.5–156.1)	147.5 (142.5–156)	0.000

Data are expressed as median (25th–75th percentile)

## Discussion

In these non-random blood samples for gas analysis, we showed that maternal O_2_ exposure at super-physiological levels (radial arterial blood PO_2_ median 150 and interquartile 142.6–156.7 mmHg) during the second stage of labor might not change the UvPO_2_. There were also no significant differences in the UvPO_2_ between different percentiles of O_2_ exposure duration, or between room air and different percentiles of O_2_ exposure duration. As far as we know, this was the first RCT that PO_2_ was compared directly between maternal arterial blood and umbilical venous blood.

In general, the maternal arterial blood PO_2_ is about 100–110 mmHg, while the umbilical venous blood PO_2_ in term newborns is about 28 mmHg [14]. Although neonatal umbilical cord venous PO_2_ levels are lower, newborns are not hypoxic. Fetal hemoglobin structure (HbF, α2γ2) is different from that of adults (HbA, α2β2), and it is easier to bind to O_2._ The concentration of HbF (16.5 g/dL) is higher than HbA (12.5 g/dL). Therefore, Fetal umbilical venous O_2_ content is at the same level as adult arterial blood O_2_ content [15, 16]. In human blood, 98.5% O_2_ is transported by means of binding to hemoglobin, and 1.5% O_2_ is transported by means of physical dissolution [12]. Maternal inhalation of O_2_ will significantly increase the PO_2_ of her arterial blood. Polvi et al. [17] found that inhaling 50% O_2_ can make the maternal PO_2_ exceed 200 mmHg, and inhaling 100% O_2_ can make the maternal PO_2_ exceed 300 mmHg in only 5 min. When PO_2_ exceeds 150 mmHg, hemoglobin has become saturated, and O_2_ that continues to increase can only be transported by means of physical dissolution [12]. Physically dissolving transportation method is very inefficient, at standard atmospheric pressure, for every one mmHg increase in PO_2_, the dissolved O_2_ per liter of blood only increases by 0.03 ml [12].

However, PO_2_ cannot be increased indefinitely to increase physical dissolution, because data from humans and experimental animals show that O_2_ at the super-physiological level would trigger oxidative stress and oxidative damage in the mother-fetus [18–20]. Khaw et al. [20] randomized 44 women undergoing spinal canal anesthesia and found that inhaling 60% O_2_ could significantly increase the lipid peroxides, a marker of oxidative stress, including maternal arterial and neonatal umbilical arterial 8-isoprostane, malondialdehyde, and hydroperoxide. It is currently inferred that oxidative stress can cause vasoconstrictive effects [21]. Smit et al. [22] systematically reviewed 60 experimental animal studies and found that excessively high PO_2_ can cause vasoconstriction in vivo and in vitro, and there was a clear dose dependent effect. Smit et al. [23] also systematically reviewed 33 clinical studies and found that excessively high PO_2_ (234–617 mmHg) can increase vascular resistance by 11–16% in healthy people and 24.6% in patients with heart failure, and can decrease cardiac output by 10.2% in healthy people, 9.6% in patients with coronary heart disease, and 15.2% in patients with heart failure.

There were only four RCTs assessed the effect of maternal prophylactic or therapeutic inhalation of O_2_ on fetal acid-base metabolism during the second stage or active period of labor. These randomised trial showed O_2_ therapy did not improve fetal acid-base metabolism and even increased the risk of fetal acidosis. Our trial randomized 443 normal women and found there were no significant differences between 2 L/min O_2_ group and room air group in the abnormal umbilical cord arterial pH values, and found in 217 non-random samples maternal O_2_ exposure at super-physiological levels (median arterial blood PO_2_ 150 mmHg) cannot increase the umbilical cord venous PO_2_ (30.2 versus 28.3 mmHg) [11]. Raghuraman et al. [24] randomized 114 women with nonreassuring FHR tracings and found there were no significant differences between 10 L/min O_2_ group and room air group in the abnormal umbilical cord arterial pH and lactate values, and they even found long durations of O_2_ exposure were associated with lower in the UvPO_2_ (32.5 versus 25.5 mmHg). Thorp et al. [25] randomized 86 normal women and found maternal 10 L/min O_2_ exposure were associated with more umbilical cord arterial pH < 7.2 events (9/41 versus 2/44), and found this O_2_ exposure cannot increase the in the UvPO_2_ (31.2 versus 29.7 mmHg). Sirimai et al. [26] randomized 160 normal women and also found more umbilical cord arterial pH < 7.2 events under maternal O_2_ exposure with no statistical difference (8/80 versus 3/80). Our results did not conflict with previous studies. In Raghuraman et al. [24] and Thorp et al. [25] trials, the O_2_ flow rate was 10 L/min and the fraction of inspired O_2_ (FIO_2_) was about 60–80%, Thorp et al. [25] showed high level O_2_ exposure had no effect on UvPO_2,_ and Raghuraman et al. [24] even showed longer high level O_2_ exposure was associated with lower UvPO_2_. The effects of super-physiological O_2_ induced vasoconstriction would reduce blood perfusion of tissue to a greater extent than the little maternal arterial O_2_ content rise [11]. In our study, the O_2_ flow rate was 2 L/min and the fraction of inspired O_2_ (FIO_2_) was about 30%, we found low level O_2_ exposure had no effect on UvPO_2_ and the duration of exposure had no effect on UvPO_2_ (Tables 2 and 3). These three showed maternal O_2_ exposure did not increase UvPO_2_.

This secondary analysis has important limitations. First, we compared the baseline characteristics of all women and there were no significant differences between most two comparisons except antepartum hemoglobin in the oxygen group (Table S2). However, radial arterial blood samples and umbilical cord blood samples were non-randomly obtained in those non-random women who voluntarily agreed immediately after delivery. Further study is very likely to have an important impact on this analysis, which provided low quality evidence with a high selection bias. Second, the PO_2_ in O_2_ group was median 150, interquartile 142.6–156.7, and range 135.1–177 mmHg. The minimum value was only 135.1 mmHg and only half women exceeded 150 mmHg in the real world. To observe the effect of super-physiological O_2_, future study should make the PO_2_ exceed at least 150 mmHg in all women in the O_2_ group.

## Conclusion

We conclude that maternal O_2_ exposure at super-physiological levels (median arterial blood PO_2_ 150 mmHg) in normal labor may not change the UvPO_2_. Further studies in the form of RCTs would be needed to assess change in UvPO_2_ with maternal supplemental oxygenation at super-physiological levels. Our data is insufficient to change practice of maternal supplementation of O_2_ at this time.

## Supplementary information

**Additional file 1: Figure S1.** Flow diagram of trial recruitment and follow up.

**Additional file 2: Table S1.** Three-tiered fetal heart rate interpretation system.

**Additional file 3: Table S2.** Baseline characteristics of all women and non-random women.

## Data Availability

The dataset used in the present study is available from the corresponding author upon reasonable request.

## Abbreviations

ACOG: American College of Obstetricians and Gynecologists
PO_2_: Arterial partial pressure of oxygen
FHR: Fetal heart rate
O_2_: Oxygen
RCT: Randomised controlled trial
UvPO_2_: Umbilical cord venous PO_2_
