# Supplementary figures and images for: Maternal oxygen exposure may not change umbilical cord venous partial pressure of oxygen: non-random, paired venous and arterial samples from a randomised controlled trial
Source: BMC Pregnancy Childbirth. 2020 Sep 4;20:510. doi: 10.1186/s12884-020-03212-3 (PMC7650259; doi:10.1186/s12884-020-03212-3)

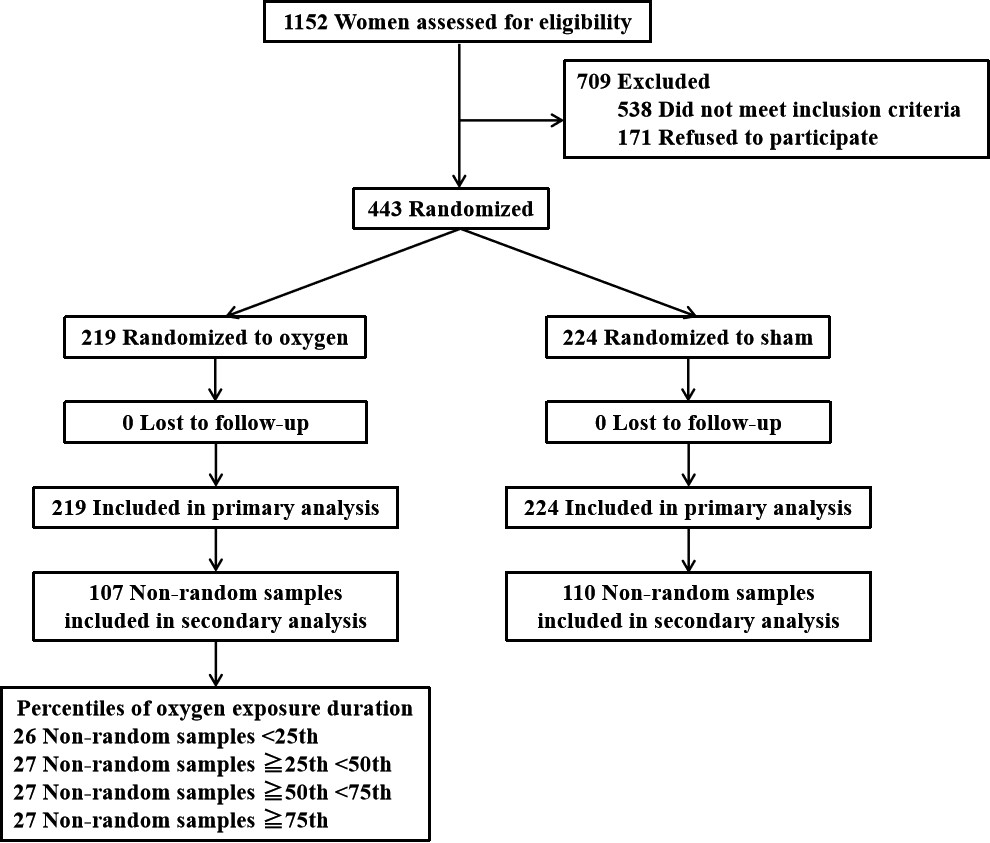

Supplement: Supplementary file 1 — Additional file 1: Figure S1. Flow diagram of trial recruitment and follow up. [file 12884_2020_3212_MOESM1_ESM.jpg]
