# Supplementary material for: Maternal oxygen exposure may not change umbilical cord venous partial pressure of oxygen: non-random, paired venous and arterial samples from a randomised controlled trial
Source: BMC Pregnancy Childbirth. 2020 Sep 4;20:510. doi: 10.1186/s12884-020-03212-3 (PMC7650259; doi:10.1186/s12884-020-03212-3)
Supplement: Supplementary file 2 — Additional file 2: Table S1. Three-tiered fetal heart rate interpretation system. [file 12884_2020_3212_MOESM2_ESM.doc]

**Table S1:** Three-Tiered Fetal Heart Rate Interpretation System

**Category I**

• Category I FHR tracings include all of the following:

• Baseline rate: 110–160 beats per minute

• Baseline FHR variability: moderate

• Late or variable decelerations: absent

• Early decelerations: present or absent

• Accelerations: present or absent

**Category II**

Category II FHR tracings includes all FHR tracings not categorized as Category I or Category III. Category II tracings may represent an appreciable fraction of those encountered in clinical care. Examples of Category II FHR tracings include any of the following:

Baseline rate

• Bradycardia not accompanied by absent baseline variability

• Tachycardia

Baseline FHR variability

• Minimal baseline variability

• Absent baseline variability with no recurrent decelerations

• Marked baseline variability Accelerations

• Absence of induced accelerations after fetal stimulation Periodic or episodic decelerations

• Recurrent variable decelerations accompanied by minimal or moderate baseline variability

• Prolonged deceleration more than 2 minutes but less than10 minutes

• Recurrent late decelerations with moderate baseline variability

• Variable decelerations with other characteristics such as slow return to baseline, overshoots, or shoulders

**Category III**

Category III FHR tracings include either

• Absent baseline FHR variability and any of the following:

—Recurrent late decelerations

—Recurrent variable decelerations

—Bradycardia

• Sinusoidal pattern

American College of Obstetricians and Gynecologists. Practice bulletin no. 116: Management of intrapartum fetal heart rate tracings. Obstet Gynecol. 2010 Nov;116(5):1232-40.

American College of Obstetricians and Gynecologists. Practice bulletin no. 106: Intrapartum Fetal Heart Rate Monitoring: Nomenclature, Interpretation, and General Management Principles. Obstet Gynecol. 2009;114(1):192-202.
