# Supplementary material for: Maternal oxygen exposure may not change umbilical cord venous partial pressure of oxygen: non-random, paired venous and arterial samples from a randomised controlled trial
Source: BMC Pregnancy Childbirth. 2020 Sep 4;20:510. doi: 10.1186/s12884-020-03212-3 (PMC7650259; doi:10.1186/s12884-020-03212-3)
Supplement: Supplementary file 3 — Additional file 3: Table S2. Baseline characteristics of all women and non-random women. [file 12884_2020_3212_MOESM3_ESM.doc]

**Table S2. Baseline characteristics**

| **Characteristic** | **Oxygen Group** | | | **Placebo Group** | | |
| --- | --- | --- | --- | --- | --- | --- |
|  | **All women (n=219)** | **Non-random women (n=107)** | ***P*** | **All women (n=224)** | **Non-random women (n=110)** | ***P*** |
| Age (y) | 29 (28-32) | 30 (27-32) | 0.052 | 29 (27-31) | 29 (28-31) | 0.071 |
| Gestational age (wk) | 40.0 (39.3-40.7) | 40.0 (39.3-40.9) | 0.773 | 40.0 (39.3-40.4) | 40.0 (39.3-40.4) | 0.062 |
| Admission BMI (kg/m^2^) | 26.5 (24.8-28.6) | 25.8 (24.5-27.6) | 0.062 | 26.6 (24.6-28.7) | 26.6 (24.5-28.6) | 0.857 |
| Antepartum hemoglobin (g/L) | 125 (119-132) | 126 (121-133) | 0.047 | 127 (119-134) | 127 (119-134) | 0.968 |
| Duration of first stage (min) | 480 (320-615) | 450 (310-590) | 0.211 | 450 (315-627.5) | 450 (300-660) | 0.355 |
| Duration of second stage (min) | 50 (33.3-74) | 47 (31-67) | 0.730 | 45 (30-70) | 42 (28-58) | 0.764 |

Data are expressed as median (25th-75th percentile).

BMI, body mass index.
